# Potential Association of Maker Expression of Low-Density Neutrophils and Their Phenotypes in Patients with Periodontitis: Control Study

**DOI:** 10.1155/2024/5498307

**Published:** 2024-06-06

**Authors:** Ali Omran Mousa, Ali Hussien Abass Al Hussaini

**Affiliations:** ^1^Department of Periodontology, College of Density, University of Baghdad, Baghdad, Iraq; ^2^Department of Oral and Maxillofacial Surgery, College of Dentistry, University of Baghdad, Baghdad, Iraq

## Abstract

**Background:**

Neutrophils play an important role in maintaining periodontal status in conditions of healthy homeostasis. They achieve their surveillance function by continuously migrating to the gingival sulcus and eradicating periodontal pathogens. In addition, neutrophils are considered an integral element in the pathogenesis of periodontal diseases. Among several neutrophil subsets, low-density neutrophils (LDN) have recently received attention and are linked with cancer, immunological, inflammatory, and infectious diseases. However, the presence, phenotypes, and potential role of LDN in the pathogenesis of periodontitis have not yet been investigated.

**Objectives:**

To investigate the presence, subsets (normal, band, suppressive, and active), and phenotypes via marker expression surface protein known as the cluster of differentiation (CD) (CD16b, CD14, CD15, and CD62L) of LDN in patients with periodontitis.

**Materials and Methods:**

The observational case-control study was conducted to estimate the potential role of LDNs in periodontitis. Venous blood and periodontal indices were obtained from 40 healthy control individuals and 60 periodontitis patients. Subsequently, CD16b, CD62L, CD14, and CD15 expression on the surface of LDN was examined by multicolor flow cytometry, and their subsets were classified as “normal” (CD16brightCD62Lbright), “bands” (CD16dimCD62Lbright), “suppressive” (CD16brightCD62Ldim), and “active” (CD16brightCD62Lnegative).

**Results:**

There was a significant difference in the expression of LDN markers for active and suppressive phenotypes, respectively, favoring periodontitis over the control group. In contrast, there were significantly higher levels of CD16b, CD62L, and CD15 (“normal”) in the control group when compared with the periodontitis group.

**Conclusion:**

LDN was associated with periodontitis as it was significantly increased in the periodontitis group in comparison with the control group and was positively correlated with all periodontal parameters. Cells from both groups of patients (periodontitis and control) expressed a normal mature phenotype (CD16b + High, CD62L + High, CD15+, and CD14-). Regarding subsets, the normal LDN (CD16brightCD62Lbright) was the most predominant phenotype in both periodontitis and control groups. However, the active subset increased in periodontitis compared to normal, indicating their destructive role in periodontitis.

## 1. Introduction

Periodontitis is an immunological inflammatory condition triggered by the body's response to a growing dental biofilm. The mechanism behind this reaction involves chemoattractants, which attract leukocytes, including neutrophils, to the site of inflammation [1–3]. Neutrophils are responsible for pathogen eradication and facilitate tissue repair by reducing inflammation [4]. Despite their beneficial roles, neutrophils also have a detrimental behavior in the pathogenesis of periodontal disease. An aberrant response to plaque appears to provoke neutrophils to generate an increased quantity of reactive oxygen species (ROS) [5] and to release proteases that cause collateral damage in the periodontal connective tissue [6]. Furthermore, an imbalance in homeostasis between ROS and antioxidant defense mechanisms has been linked to the development of periodontitis [7].

This functional diversity has been attributed to the wide variety of neutrophil phenotypes that are recruited during the course of the disease. Phenotype refers to the surface characteristics of a particular cell illustrated by a panel of CD makers. Subsets refer to the cells with a specific function that characterizes them over the cells of the same type [8]. Neutrophil phenotypes are subsets of neutrophils that have gained or lost the ability to perform a particular function not shared with other cell phenotypes [9].

Among the various neutrophil subtypes, low-density neutrophils (LDN) gain much interest because of their appearance in many different pathological conditions [10, 11]. They represent a unique subpopulation of neutrophils (with specific functional characteristics) that increase in numbers as disease severity progresses [11, 12]. Previous research has shown the presence of LDN in the peripheral blood of systemically healthy subjects, with a prevalence ranging from 2 to 10% of total peripheral blood leukocytes [13, 14].

The potential role of LDN in the pathogenesis of periodontitis is still unclear. However, it is important to investigate the expression of markers on the cell surface and the physical properties, buoyancy, and nuclear morphology of LDN to identify their role in different diseases [15]. In the current study, the expression of a panel of surface markers (CD16b, CD62L, CD15, and CD14) has been used to assess the phenotype of LDN and to determine their subsets classified as “normal” (CD16brightCD62Lbright), “bands” (CD16dimCD62Lbright), “suppressive” (CD16brightCD62Ldim), and “active” (CD16brightCD62Lnegative) in systemically healthy subjects with or without periodontitis. We emphasize that low-density neutrophils are unique neutrophil phenotypes that are associated with various systemic and infectious conditions, whose specific behavior in periodontitis has not previously been studied.

### 1.1. Study Hypothesis

#### 1.1.1. Null Hypothesis

There are no statistically significant differences in the presence, phenotypes, and marker expression of low-density neutrophils (LDNs) between patients with periodontitis and healthy controls.

## 2. Materials and Methods

### 2.1. Study Design

This observational case-control study was conducted at the College of Dentistry, University of Baghdad; the Iraqi Blood Transfusion Center; Husseinya Specialized Dental Center; and teaching laboratories in the Medical City (PCR lab) from August 2022 to March 2023.

### 2.2. Ethical Approval

All procedures included in this study were conducted according to the Helsinki Declaration of 1964 and its later amendments for human research. The ethics committee approved the protocol, College of Dentistry, University of Baghdad (reference number: 450; project number: 450622; date: 19\1\2022). Each patient was asked to sign an informed consent form after receiving an explanation about the nature and aims of the study. After signing the consent form, a clinical examination was carried out and a blood sample was collected.

### 2.3. Inclusion Criteria

A total of 100 systematic individuals were recruited to participate in the study with at least 20 natural teeth and were divided into two groups: a control group with a healthy periodontium (Ctrl, *n* = 40) with a mean age of 34.5 years ranging (from 20 to 56 years) and a periodontitis group (P, *n* = 60) with a mean age of 36.3 years ranging (from 21 to 57 years). The case definitions for each group were, for the healthy periodontium group, bleeding on probing (BOP) less than 10%, probing pocket depth (PPD) less than or equal to 3 mm, and no observed clinical attachment loss (CAL) (intact periodontium) [16]. The participants with periodontitis were selected based on the presence of CAL at the interproximal sites of two nonadjacent teeth or a CAL of at least 3 mm on the outer (facial) or inner (lingual/palatal) surfaces of the ≥2 teeth, together with a pocket depth of >3 mm in at least two teeth. In addition, all periodontitis cases were “unstable,” defined as a condition of the presence of BOP on 4 mm PPD or PPD ≥ 5 mm [17].

### 2.4. Exclusion Criteria

**Figure 1:**
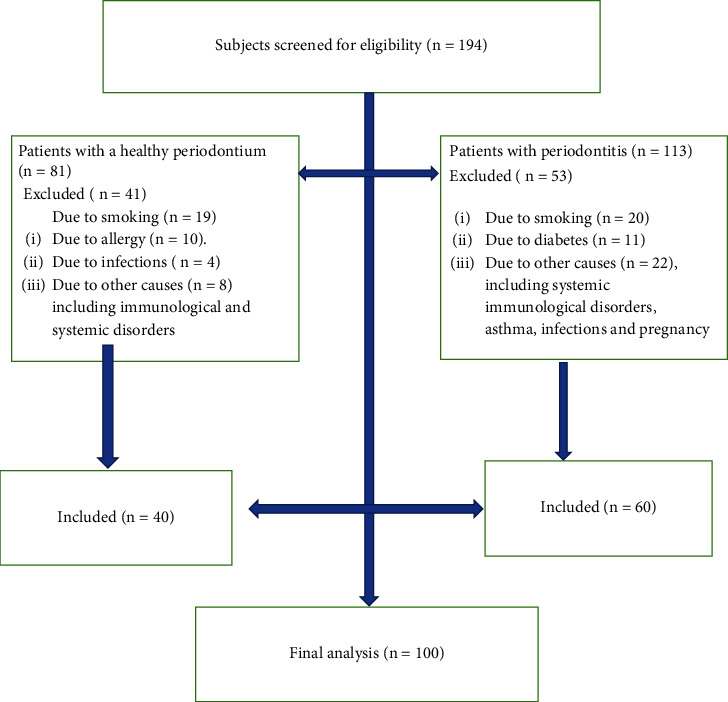
Flowchart of the study.

Individuals with a dental implant(s) or who were suffering from a systemic or oral autoimmune, infectious, or inflammatory disease were excluded, as were pregnant women, smokers, those receiving medications within the last three months (steroids and anti-inflammatory drugs 48 hr after the last dose), and those with endocrine disorders. Regarding periodontal parameters, wisdom teeth were excluded from the examination (Figure 1).

### 2.5. Pilot Study

A pilot study used the first 12 samples collected from each group (control and periodontitis). A total of 24 samples were analyzed in the laboratory by flow cytometry to validate the method of LDN purification required for cell labeling, ROS production, and phagocytic activity. The values obtained from this pilot study were added to the final data of the main study.

### 2.6. Sample Size

Levels of one of the biomarkers (CD16b) obtained from the pilot study (12 samples from each of the control and periodontitis groups) were assigned to calculate the sample size for this study using G-Power 3.1.9.4. The study was designed to have a 90% power and a type I error probability *α* = 0.05 and a large effect size (0.8) between the two groups (the actual effect size being 1.5 according to means/SD derived from the pilot study). A sample size of 34 samples/group was calculated and rounded up to 40 samples/group.

### 2.7. Calibrations

Calibration sessions were performed before the start of the study between the main investigator and a gold-standard periodontics specialist. Inter- and intraexaminer calibration for categorical variables (PI and BOP) were assessed by using a kappa coefficient test. The targeted level was a kappa value of ≥75% to ensure a good level of agreement. For continuous variables (PPD and CAL), the level of agreement rounded to the nearest millimeter should be >0.9 as determined by an interclass coefficient test. These sessions were conducted on five patients, and the results were discussed. If a high degree of inconsistency was present, then these discrepancies were discussed and the session was repeated.

### 2.8. Clinical Examination

The clinical parameters for all the existing dentitions included full-mouth PI [18], full-mouth BOP, PPD, and CAL.

### 2.9. Blood Collection Procedure

An oral examination was performed, and 10 ml of blood was collected and immediately placed in a 15 ml tube containing a 5 mM concentration of the dipotassium salt of ethylenediaminetetraacetic acid (EDTA). It was stored for 30 minutes at a temperature of 4°C while shielding from light. The LDN were purified within a period not exceeding 6 hours to avoid cell death or any change in marker expression and LDN phenotype.

### 2.10. Low-Density Neutrophil Purification

Isolation of LDN was performed by combining 10 ml of anticoagulated blood with 2 ml of 6% dextran T500, gently mixing the solution, and allowing it to settle for 45 minutes to let the red blood cells sink to the bottom. Subsequently, the leukocyte-rich plasma was added over 5 ml of Lympho-Paque at a density of 1.077 g/ml. It was then centrifuged at 520 × g for 20 min at 4°C using a Thermo Scientific ultrarefrigerated cold Megacentrifuge 8R. After centrifugation, a layer of mononuclear cells (MNC) containing monocytes, lymphocytes, and LDN was located in the zone between the plasma and the Lympho-Paque. In addition, a pellet of normal, high-density neutrophils was located in the lowermost part of the tube. MNC containing LDN was collected; diluted with phosphate buffered saline (PBS), a buffer solution (pH ~7.4) commonly used in biological research (dilution factor 1 : 2); and kept on ice until used.

### 2.11. Low-Density Neutrophils: Phenotyping and Multicolor Flow Cytometry Analysis

**Figure 2:**
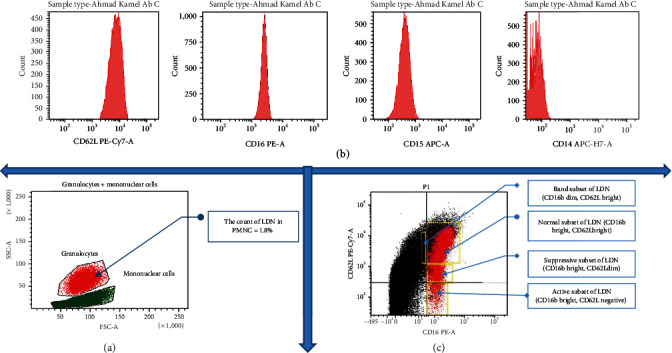
(a) Gating strategies, marker expression, and LDN phenotyping in the control group. (b) Identification of LDN depended on forward scatter acquisition (FSC-A) and side scatter acquisition (SSC-A). (c) A combination of markers of the expression of CD16b, CD62L, CD15, and CD14. LDN was used for phenotyping bands (CD16dimCD62Lbright), normal (CD16brightCD62Lbright), suppressive (CD16brightCD62Ldim), and active (CD16brightCD62Lnegative).

**Figure 3:**
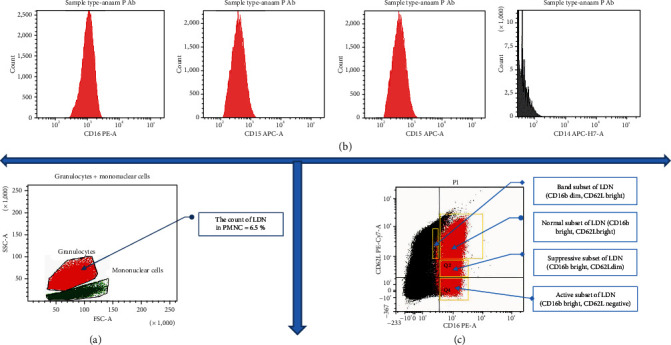
(a) Gating strategies, marker expression, and LDN phenotyping in the periodontitis group. (b) Identification of LDN depended on FSC-A and SSC-A. (c) A combination of markers of the expression of CD16b, CD62L, CD15, and CD14. LDN was used to phenotype bands (CD16dimCD62Lbright), normal (CD16brightCD62Lbright), suppressive (CD16brightCD62Ldim), and active (CD16brightCD62Lnegative).

The detection of surface-fluorescent antibodies was performed by flow cytometry following the method described by García-García et al. [19]. Briefly, a total of 1 × 10^6^ cells were labeled for 30 minutes at 4°C in the dark in a 1.5 ml Eppendorf tube with the PE Mouse IgG2a anti-human CD16b antibody, APC anti-human CD14 antibody (0.25 *μ*g/ml), PE/Cyanine5 APC anti-human CD15 antibody (1/40 dilution), and anti-human CD62L antibody (0.25 *μ*g/ml) in 100 *μ*l of labeling buffer (PBS + 1% bovine serum albumin (BSA) + 0.1% NaN3). Cells were washed once with 1 ml of PBS and then fixed in 1% paraformaldehyde. The cells were examined using a BD FACSCanto II multicolor flow cytometer equipped with three lasers: a blue laser (488 nm, air-cooled, 20 mW solid-state), a red laser (633 nm, 17 mW HeNe), and a violet laser (405 nm, 30 mW solid-state). It provides rapid, multiparametric analysis of various cellular subpopulations of interest. Its applications include immunology, immunooncology, virology, and immune monitoring. BD Biosciences has developed innovative dyes across every laser line to provide panel design flexibility and a comprehensive portfolio of conjugated antibodies to help characterize surface, intracellular, or secreted markers deeply. Cells were gated by dot-plot analysis, and a total of 10,000 cells were gathered for each sample. Subsequently, data were processed using the BD FACSDiva™ Software v9.0. (Figures 2 and 3).

### 2.12. Statistical Analysis

The data were analyzed for normal distribution using the Shapiro-Wilk test at *p* < 0.05. Spearman's tests were used to detect correlations with the periodontal parameters in each group. The Mann–Whitney *U* test compared the control, periodontitis, and other variables. The level of significance was set at *p* < 0.05 for all variables. Statistical analysis was performed in SPSS (v25, IBM SPSS Statistics for Windows, I.B.M. Corp.).

## 3. Results

### 3.1. Expression of CD16b, CD62L, CD14, and CD15 on Low-Density Neutrophils

Flow cytometric analysis illustrates that LDN in the control group represented about 2.2% of the total peripheral mononuclear cell layer. However, in periodontitis, LDN represented about 3.4% of the total peripheral mononuclear cell layer.

**Figure 4:**
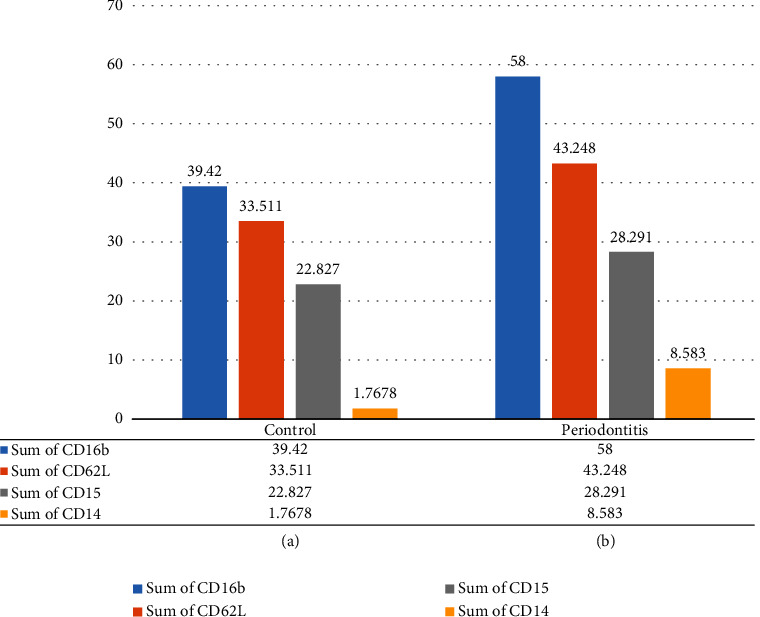
Expression of markers on the surface of LDN: (a) in the control group and (b) in the periodontitis group.

**Figure 5:**
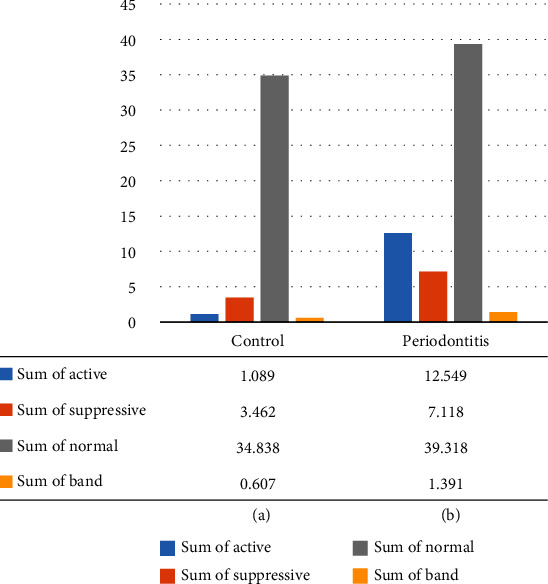
Distribution of LDN phenotypes: (a) in the control group and (b) in the periodontitis group.

In the control group, about 96.7% of LDN expressed CD16b, 83% expressed CD62L, 57% expressed CD15, and only 4% of LDN expressed CD14. Consequently, the phenotype of LDN in the control group was characterized by the expression of CD16b + High, CD62L + High, CD15+, and CD14- (Figure 4(a)). On the other hand, in the periodontitis group, the expression of CD markers on the surface of LDN revealed that 98.5% of LDN expressed CD16b, 72% expressed CD62L, 47% expressed CD15, and only 14% expressed CD14. As a result, the phenotype of LDN in periodontitis was CD16b + High, CD62L + High, CD15 +, and CD14- (Figure 4(b)). Consequently, LDN expresses a normal mature phenotype in both the control and periodontitis groups (Figures 5(a) and 5(b)).

Regarding subsets, the normal subset was the most prevalent in both groups (periodontitis and control), accounting for 87% of the total LDNs in the control group and 65% in the periodontitis group. In the control, the suppressive LDNs held the second-most predominant subset at 8.7% of total LDNs. Consequently, active represents about 21% and holds the second most predominant subset in the periodontitis group. The third most predominant subset in the control group was the active LDN at 2.7%; however, it was suppressive at 11% in the periodontitis group. The band subset is the least existing subset in both study groups, at 1.5% in the control group and at 2.3% in the periodontitis group.

### 3.2. Intergroup Comparison

A comparison showed a significantly higher BOP and PI in the periodontitis group than in the control group (Table 1). Furthermore, there were significantly higher levels of LDN, CD16b, CD14, and active and suppressive LDN in the periodontitis compared to the control groups. On the other hand, there were significantly higher levels of CD62L, CD15, and the normal subset of LDN in the control compared to the periodontitis group (*p* < 0.05), respectively.

### 3.3. Correlations

#### 3.3.1. In the Control Group

CD16b correlated significantly and positively with BOP and negatively with band LDN.

The band subset was positively correlated with suppressive and active and negatively correlated with BOP and normal. The normal subset of LDN was significantly negatively correlated with suppressive and active. The suppressive subset was significantly positively correlated with the active (Table 2).

#### 3.3.2. In the Periodontitis Group

BOP was correlated positively with LDN and CD62L and negatively with suppressive LDN. There were significant positive correlations between PI and LDN, CD62L, and active LDN. On the other hand, PI was negatively correlated with the band and suppressive LDN. However, PPD was positively correlated with LDN and CD62L. CAL was correlated significantly and positively with LDN and CD62L. CD16b was correlated significantly and positively with BOP, PI, CAL, and CD62L. LDN was positively correlated with all periodontal parameters, including CD62L. Active LDN correlated negatively with both normal and suppressive LDN. Suppressive LDN correlated significantly and positively with band LDN (Table 3).

## 4. Discussion

This study showed that LDN was consistently present in both groups, with a significant increase in the periodontitis group compared to the healthy periodontium, suggesting a potential role of LDN in the pathogenesis of periodontal disease. This finding coincided with a significant association between the percentage of LDN and periodontal parameters (BOP, PPD, PI, and CAL) in the periodontitis group. Indeed, estimation of the bleeding on probing (BOP) is the most efficient method for determining the health or inflammation of the gingival tissues [20], and previous prospective studies concluded that BOP was a reliable clinical sign for measuring the severity or stability of periodontal disease [21, 22]. Hence, the observed positive correlation between the BOP and the proportion of LDN in individuals with periodontitis suggests that an increase in periodontal disease severity was associated with an increased LDN count.

In addition, the simultaneous increase in dental biofilm and pocket depth might lead to a noticeable rise in subgingival pathogen levels [23–25], which may promote the systemic spread of the periodontal pathogens and their products via the ulcerated pocket epithelium [26, 27], which may recruit more LDN and worsen the periodontal condition [12]. This would explain the observed positive correlation of LDN with PI and PPD.

The current data revealed that LDN exhibits a normal mature phenotype in both the control and periodontitis groups, characterized by expression of CD16b + High, CD62L + High, CD15+, and CD14-. This finding is consistent with those of Scapini et al. on healthy individuals [10]. In fact, the increase in the normal subset reflects the improvement of periodontal health. A recent longitudinal study on periodontitis patients illustrated that normal subsets gradually increased upon progressing periodontal treatment and improving periodontal health [28].

The suppressive subset held second place in the control group and was the third most prominent phenotype in the periodontitis group. Moreover, the proportion of suppressive LDN correlated negatively with BOP in the periodontitis group. They appear to perform a regulatory function that is potentially required to limit the damaging effects of excessive T cell activation in the bloodstream, triggered by bacterial burden, and to maintain the compartmentalization between the peripheral circulation and the periodontium [29]. The intergroup comparison revealed that the suppressive subset statistically increased in the periodontitis group compared to the control group. These outcomes were supported by a prior longitudinal study on periodontitis patients, which found that the count of suppressive LDN decreased gradually upon progressing periodontal treatment [28].

In contrast, the active LDN subset was the second most prevalent subset in the periodontitis group and was positively correlated with BOP. The active phenotype is continuously recruited to the periodontal sites [24]. However, under the burden of *P. gingivalis* and other periodontal pathogens, the active neutrophils are recruited to the periodontium, which is characterized by the release of ROS, cationic peptides, and enzymes such as MMP-9 (gelatinase B) and matrix metalloproteinase-8 ((MMP-8) neutrophil collagenase). All of the aforementioned events contribute to an increased level of damage to the periodontal tissue [25]. Furthermore, the activation of these LDNs may be attributed to the entrance of periodontal pathogens via the ulcerated pocket epithelium observed in periodontitis [26, 27].

Moreover, a comparison between the two groups showed that the average level of the normal LDN was higher in the control group than in the periodontitis group. On the other hand, both active and suppressive phenotype levels were elevated in individuals with periodontitis as compared to the control group. Indeed, in periodontitis, both bacteria [29] and the inflammatory response [30] lead to LDN becoming activated and shedding CD62L [31], making them lose their normal phenotype and switch to the active or suppressive phenotype. Additionally, the shedding of CD62L has been suggested as a potential mechanism for neutrophil deadhesion, which is essential for detachment from endothelial cells before transmigration into sites of inflammation [32].

The percentage of the band phenotype was relatively low in both the control and periodontitis groups and had a reciprocal relation with the normal phenotype. No difference was observed between the groups regarding the band phenotype.

Band neutrophils can be identified via their band- or sausage-like nuclei since they are immature neutrophils at the final stage of maturation. It might be challenging to distinguish mature (segmented) neutrophils from band neutrophils based on morphology [33].

Hence, CD marker expression has been used to assess the band LDN population exhibiting a CD16b dim, CD62Lbright phenotype [28]. An increase in the proportion of unsegmented band neutrophils in the circulation has been described as a left shift, a process documented by Marsh et al. [34].

During neonatal sepsis [35] or severe bacterial infection [36], many mature neutrophils in the bloodstream or marginal pool transmigrate and are consumed at sites of infection. This leads to gradual depletion in neutrophil number, which induces the bone marrow to pump the stored band neutrophils into blood vessels and produce a new neutrophil clone to compensate for the neutrophil depletion until the infection has been eliminated [33].

CD16b showed a high expression in both study groups, with a slightly enhanced expression in periodontitis compared to the control. Moreover, CD16b was correlated positively with all periodontal parameters (BOP, PI, PPD, and CAL) in both study groups. The explanation of CD16b's positive correlations with periodontal parameters is that it is involved in the detection and phagocytosis of bacteria opsonized by IgG. Indeed, CD16b is an Fc receptor exclusively expressed on the surface of human neutrophils to facilitate the recognition and phagocytosis of IgG-opsonized pathogens. This process leads to the activation of neutrophils, the initiation of the proinflammatory pathway, and the synthesis and secretion of antimicrobial agents [32]. Furthermore, the observed effector functions following the cross-linking of CD16b encompass actin filament assembly [37], degranulation [38], phagocytosis [39], activation of respiratory burst [40, 41], and the recruitment of neutrophils in immune complex-mediated inflammation [42].

CD62L (L-selectin) was highly expressed on the surface of LDN in both study groups, favoring the control group. Previous *in vitro* studies suggested that L-selectin undergoes rapid removal from the plasma membrane of neutrophils through ectodomain shedding [43].

The shedding of CD62L is a well-established indicator of neutrophil activation or partial activation (priming). However, the presence of a hyperactive neutrophil phenotype (CD62L-) is widely recognized as a key factor in the development of chronic periodontitis [5].

Moreover, a positive correlation was observed between CD62L (L-selectin) and BOP (a primary indicator of inflammation) [44, 45]. A related study demonstrated that the expression of CD62L was higher during active bone marrow release [46] induced by an inflammatory process [44, 47]. Consequently, upon inflammation, the total number of inflammatory cells increases [48], including LDN, regardless of their phenotypes, mostly expressing CD62L+, except active phenotypes expressing CD62L-.

The results of the current study demonstrate that LDN expresses CD14- and CD15+ in both groups. The observed phenotype is a distinguishing characteristic of LDN, setting it apart from monocytes that exhibit a different expression pattern, CD14+ and CD15- [29, 45].

The fucosyl-N-acetyl-lactosamine (Le(X)) or CD15) is a carbohydrate adhesion molecule that binds to glycoproteins, glycolipids, and proteoglycans on the leukocyte cell membrane. CD15 facilitates phagocytosis and chemotaxis and is often used as a marker in hematologic malignancies [30].

In the current study, the expression of CD15 on LDNs was significantly higher in the control group compared to the periodontitis group. This finding aligns with a prior study that demonstrated an elevation in the expression of CD15 on neutrophils in the healthy control group compared to both tuberculosis (T.B.) patients undergoing treatment and the active T.B. group [30, 49].

Moreover, the level of CD15 expression was elevated on the surface of neutrophils in T.B. patients who had been successfully treated compared to those who still had active T.B. In addition, the count of neutrophils was negatively correlated with CD15 expression in all study groups [30].

These findings support the result of the present study of decreasing LDN count and upregulation of CD15 expression in the control group. Conversely, in periodontitis, the count of LDNs was elevated, and CD15 expression was downregulated.

CD14 is considered a human monocyte identification antigen and a pattern recognition receptor (PRR) of innate immunity (triggers monocyte intracellular reactions in response to bacterial interactions) [50].

The outcomes of the present study revealed that LDNs express the CD14- phenotype in both study groups. This outcome agreed with prior research demonstrating that the identification of neutrophils was determined by using an FSC/SSC granulocyte gate, excluding doublets based on SSC height/width, and evaluating CD14- expression [51].

The analysis of site-specific neutrophils purified from saliva, gingival crevicular fluid (GCF), and junctional epithelium can offer a more comprehensive understanding of the particular cell subsets and their role in the pathogenesis of periodontitis. However, a prior investigation has demonstrated that neutrophils from diseased periodontal sites exhibit more gene alterations than those in healthy periodontium [52]. Furthermore, in humans, there is no specific antigen that can distinguish normal-density neutrophils (NDN) from low-density neutrophils (LDN) [53]. This makes the purification of LDN from the peripheral blood via density gradient centrifugation and flow cytometry analysis the sole method available for studying LDN.

Multiple factors might influence the expression of extracellular surface markers on neutrophils. For instance, previous studies have shown an elevation in CD62L expression during active bone marrow release [46]. However, the process of neutrophil aging [54], as well as the administration of certain medicines, such as nonsteroidal and anti-inflammatory drugs [55] and steroids [56], have been associated with a reduction in surface CD62L levels [28].

A prior investigation has shown that the administration of steroids and anti-inflammatory drugs at a dosage of 2.0 mg/kg intravenously resulted in a reduction in CD62L expression on circulating neutrophils within 12 to 24 hours after treatment. The expression levels returned to their original levels within 48 hours. In addition, dexamethasone reduced CD62L expression on segmented neutrophils in the bone marrow but did not have the same effect on those already in circulation [57].

Regarding the data collected for the current study, the case-control design is considered a limitation. The production, activation, priming, and clearance of neutrophils is a highly sensitive process and may be affected by many environmental and systemic factors [58]. Thus, a more detailed study with a larger sample size, more follow-up time, and an expanded set of CD markers is needed to evaluate the potential impact of LDN on the development of neuropathy.

The null hypothesis was rejected, as the results of the present study revealed that LDN increased in the peripheral blood of periodontitis patients compared to controls and positively correlated with all periodontal parameters. It exhibited the normal phenotype but with a predilection toward the active phenotype, indicating the probable role of LDN in the pathogenesis of periodontitis. Nevertheless, the precise function they serve remains ambiguous and requires more examination.

### 4.1. Identification of New Biomarkers

Understanding the behavior and functional properties of LDN in periodontitis provides valuable insights into the disease process. This may help to identify specific biomarkers associated with disease severity or progression and facilitate early diagnosis and personalized treatment.

## 5. Conclusion

This study showed that LDN increased significantly in systemically healthy patients with periodontitis compared to controls and was positively associated with all periodontal parameters. The normal phenotype (CD16bright, CD62Lbright) was the most prevalent in both study groups. In the periodontitis group, there was an increase in the active phenotype (CD16bright, CD62Lnegative) compared to the control group. The active phenotype was positively associated with all periodontal parameters.

### 5.1. Research Rationale

Neutrophils are major immune cells involved in protection against tissue infection and the resolution of inflammation. Low-density neutrophils (LDNs) are a distinct subset of neutrophils associated with systemic inflammatory conditions. These cells exhibit different behaviors and functions compared to conventional neutrophils. Thus, it is important to investigate the role of LDNs in periodontal disease for several reasons:

Immune mechanisms in periodontitis: studying LDNs can shed light on the complicated interaction among the immune system and the pathogenesis of periodontitis. It can help perceive capacity targets for remedy or prevention techniques through information on the position of LDNs inside the inflammatory reaction and tissue destruction.

Unmet scientific want: periodontitis is a persistent inflammatory circumstance that affects a good sized proportion of the population. Despite advances in periodontal remedy, there is nevertheless a want for improved remedy modalities and preventive techniques. Investigating LDNs in periodontitis may additionally lead to the development of novel healing interventions.

By analyzing the expression of CD16b, CD14, CD15, and CD62L on LDNs in periodontitis sufferers and comparing it to a managed institution, this observation is aimed at contributing to the information of the immune mechanisms worried in periodontitis and doubtlessly discovering new goals for treatment or prevention techniques.

The research purpose must be concise and absolutely articulate the importance of the observation and its potential contribution to the sector. It also needs to highlight any gaps in expertise that the study aims to address.

## Figures and Tables

**Table 1:** Intergroup comparison between the study groups.

Biomarker	Mean control	Mean periodontitis	*p* value^∗^
Age	34.48	36.28	0.430
BOP (%)	0.0683	0.4050	0.000^∗^
PI	0.3717	1.283	0.000^∗^
LDN in PMNC (%)	2.2	3.4	0.007^∗^
CD16b (%)	96.7	98.6	0.006^∗^
CD62L (%)	83.8	72.1	0.013^∗^
CD15 (%)	57.1	47.1	0.032^∗^
CD14 (%)	4.4	14.3	0.020^∗^
Band (%)	1.5	2.3	0.199
Normal (%)	87.1	65.5	0.000^∗^
Suppressive (%)	8.7	11.9	0.048^∗^
Active (%)	2.7	20.9	0.002^∗^

*p* value ≤ 0.05. ^∗^Significant.

**Table 2:** The correlation among the variables in the control group.

	Spearman correlation	Age	BOP (%)	PI	LDN (%)	CD16b (%)	CD62L (%)	CD15 (%)	CD14 (%)	Band (%)	Normal (%)	Suppressive (%)	Active (%)
Age	Correlation coefficient	1.000											
	Sig. (2-tailed)												
BOP (%)	Correlation coefficient	0.052	1.000										
	Sig. (2-tailed)	0.748											
PI	Correlation coefficient	-0.131	0.689^∗∗^	1.000									
	Sig. (2-tailed)	0.419	0.000										
LDN (%)	Correlation coefficient	0.233	-0.009	0.104	1.000								
	Sig. (2-tailed)	0.147	0.956	0.525									
CD16b (%)	Correlation coefficient	-0.181	0.633^∗∗^	0.433^∗∗^	-0.244	1.000							
	Sig. (2-tailed)	0.264	0.000	0.005	0.129								
CD62L (%)	Correlation coefficient	-0.032	-0.134	-0.246	0.033	-0.065	1.000						
	Sig. (2-tailed)	0.844	0.409	0.127	0.839	0.691							
CD15 (%)	Correlation coefficient	-0.179	-0.024	-0.177	-0.105	-0.115	0.073	1.000					
	Sig. (2-tailed)	0.269	0.885	0.274	0.521	0.480	0.653						
CD14 (%)	Correlation coefficient	-0.191	0.158	0.032	-0.247	0.008	0.057	0.191	1.000				
	Sig. (2-tailed)	0.239	0.331	0.847	0.125	0.960	0.726	0.237					
Band (%)	Correlation coefficient	-0.138	-0.325^∗^	-0.243	-0.002	-0.322^∗^	0.005	-0.042	0.168	1.000			
	Sig. (2-tailed)	0.397	0.041	0.131	0.989	0.043	0.976	0.799	0.301				
Normal (%)	Correlation coefficient	-0.031	0.065	0.134	-0.012	0.157	0.085	0.048	-0.158	-0.613^∗∗^	1.000		
	Sig. (2-tailed)	0.848	0.689	0.410	0.942	0.332	0.602	0.770	0.329	0.000			
Suppressive (%)	Correlation coefficient	0.062	-0.006	-0.064	0.105	-0.172	-0.068	0.045	0.082	0.427^∗∗^	-0.941^∗∗^	1.000	
	Sig. (2-tailed)	0.702	0.972	0.694	0.520	0.287	0.675	0.781	0.616	0.006	0.000		
Active (%)	Correlation coefficient	0.019	-0.131	-0.195	-0.242	-0.075	0.005	-0.217	0.259	0.462^∗∗^	-0.733^∗∗^	0.554^∗∗^	1.000
	Sig. (2-tailed)	0.907	0.421	0.228	0.132	0.646	0.974	0.179	0.107	0.003	0.000	0.000	

^∗^Correlation is significant at the 0.01 level (2-tailed). ^∗∗^Correlation is significant at the 0.05 level (2-tailed).

**Table 3:** The correlation among the variables in the periodontitis group.

	Spearman correlation	Age	BOP (%)	PI	PPD	CAL	LDN (%)	CD16b (%)	CD62L (%)	CD15 (%)	CD14 (%)	Band (%)	Normal (%)	Suppressive (%)	Active (%)
Age	Correlation coefficient	1.000													
	Sig. (2-tailed)														
BOP (%)	Correlation coefficient	0.141	1.000												
	Sig. (2-tailed)	0.284													
PI	Correlation coefficient	0.175	0.808^∗∗^	1.000											
	Sig. (2-tailed)	0.180	0.000												
PPD	Correlation coefficient	0.303^∗^	0.808^∗∗^	0.653^∗∗^	1.000										
	Sig. (2-tailed)	0.019	0.000	0.000											
CAL	Correlation coefficient	0.176	0.492^∗∗^	0.442^∗∗^	0.532^∗∗^	1.000									
	Sig. (2-tailed)	0.179	0.000	0.000	0.000										
LDN (%)	Correlation coefficient	0.193	0.892^∗∗^	0.862^∗∗^	0.702^∗∗^	0.452^∗∗^	1.000								
	Sig. (2-tailed)	0.139	0.000	0.000	0.000	0.000									
CD16b (%)	Correlation coefficient	0.043	0.730^∗∗^	0.681^∗∗^	0.643^∗∗^	0.384^∗∗^	0.665^∗∗^	1.000							
	Sig. (2-tailed)	0.743	0.000	0.000	0.000	0.002	0.000								
CD62L (%)	Correlation coefficient	0.154	0.614^∗∗^	0.616^∗∗^	0.604^∗∗^	0.425^∗∗^	0.575^∗∗^	0.497^∗∗^	1.000						
	Sig. (2-tailed)	0.240	0.000	0.000	0.000	0.001	0.000	0.000							
CD15 (%)	Correlation coefficient	-0.119	-0.018	0.109	0.037	0.097	0.045	-0.015	0.005	1.000					
	Sig. (2-tailed)	0.365	0.891	0.406	0.781	0.461	0.730	0.909	0.972						
CD14 (%)	Correlation coefficient	-0.060	-0.029	0.049	0.103	-0.004	-0.004	0.116	0.072	0.166	1.000				
	Sig. (2-tailed)	0.649	0.826	0.712	0.434	0.973	0.977	0.378	0.587	0.206					
Band (%)	Correlation coefficient	-0.211	-0.056	-0.265^∗^	0.047	0.002	-0.196	-0.046	0.106	-0.081	-0.054	1.000			
	Sig. (2-tailed)	0.106	0.669	0.041	0.721	0.989	0.134	0.724	0.421	0.541	0.684				
Normal (%)	Correlation coefficient	-0.038	-0.011	-0.078	-0.110	0.090	-0.037	-0.157	0.083	0.018	-0.052	-0.146	1.000		
	Sig. (2-tailed)	0.775	0.931	0.552	0.404	0.492	0.778	0.230	0.529	0.894	0.694	0.266			
Suppressive (%)	Correlation coefficient	-0.177	-0.288^∗^	-0.303^∗^	-0.060	-0.150	-0.326^∗^	-0.216	-0.072	-0.009	0.007	0.525^∗∗^	-0.062	1.000	
	Sig. (2-tailed)	0.175	0.025	0.019	0.648	0.253	0.011	0.098	0.586	0.944	0.960	0.000	0.636		
Active (%)	Correlation coefficient	0.045	0.177	0.259^∗^	0.114	-0.075	0.198	0.184	0.090	0.009	0.144	-0.156	-0.704^∗∗^	-0.303^∗^	1.000
	Sig. (2-tailed)	0.735	0.175	0.046	0.385	0.568	0.130	0.158	0.494	0.947	0.271	0.233	0.000	0.019	

## Data Availability

The data generated and analyzed in the present study can be provided upon proper request.
